# ’Stuck or unstable’ — partnerships between GPs and patients with complex chronic conditions: A qualitative study

**DOI:** 10.3399/BJGPO.2024.0146

**Published:** 2025-06-04

**Authors:** Mads Aage Toft Kristensen, Mette Bech Risør, Andreas Søndergaard Heltberg, Tora Grauers Willadsen, Ann Dorrit Guassora

**Affiliations:** 1 Centre for General Practice, The Research Unit for General Practice in Copenhagen, Slagelse and Køge and Section for General Practice, Department of Public Health, University of Copenhagen, Copenhagen, Denmark; 2 General Practice Research Unit, Department of Community Medicine, UiT The Arctic University of Norway, Tromsø, Norway

**Keywords:** Comorbidity, General practice, Patient perspectives, Qualitative research

## Abstract

**Background:**

In chronic care, patient–GP collaboration is essential, but might be challenging if patients have complex health problems because of multimorbidity, psychosocial predicaments, and addiction problems. To understand and manage these challenges, it is important to explore how patients and GPs attempt to collaborate, to maintain and achieve an alliance in order to gain good quality of care.

**Aim:**

To explore how dyads of GPs and patients, who GPs deem have complex health problems and difficulties following treatment, perceive and manage challenges in their chronic care partnership.

**Design & setting:**

This was a qualitative study from Danish general practice in deprived, rural areas.

**Method:**

Semi-structured interviews were conducted with 12 dyads of GPs and patients with doctor-assessed complex chronic conditions and difficulties following treatment. The principles of systematic text condensation were used in the analysis.

**Results:**

Overall, the patient–GP collaboration could be characterised as either ‘stuck’ or ‘unstable’. In both types, the challenges were identified as pointless consultations, conflicts about lifestyle, resignation, concealment of information, and hopelessness. These challenges could be managed by solving conflicts, adjusting to the patient’s needs, accommodating the challenges in the relationship, and offering continued emotional support even with unsolved medical problems.

**Conclusion:**

Care of patients with complex health problems may present several challenges. In this study, patients and GPs experienced the relational dimension as crucial for collaboration. A robust therapeutic alliance, incorporating the patient’s agenda, offers an essential foundation for enhancing care in individuals with complex health problems.

## How this fits in

The chronic care model (CCM) emphasises the collaboration between the patient and the GP, but little is known about how the many patients with complex chronic conditions and their GPs experience the challenges and partnerships in the attempt to collaborate and maintain an alliance. This Danish qualitative study of dyads of patients and GPs found that despite unsolved biomedical problems the GP can make an important difference to the patient by solving conflicts, adjusting to the patient’s needs, providing continued support, and being open to the exploration of emotional problems. Development and testing of new initiatives for more proactive and empowering health care for patients with complex chronic conditions is needed; this remains an insufficiently implemented part of the CCM.

## Introduction

Worldwide, chronic care (CC) has been standardised in the form of disease management programmes (DMPs) to improve the quality of care and optimise the utilisation of healthcare resources. Inspired by the chronic care model (CCM),^1^ Danish DMPs emphasise the collaboration between the patient and the GP in CC.^2^

Almost the entire Danish population is registered with a tax-financed GP for primary health care, which is free at the point of use. GPs are private entrepreneurs regulated through collective agreements between the Danish regions and the organisation of GPs.^3^ Patients need referrals from their GPs to consult hospital specialists and to access municipal educational self-care support. Consequently, GPs in Denmark act as gatekeepers to other health services and play a key role in chronic care, which is organised through disease management programmes.

In 1956, CC collaboration was already described as one of the basic models of the doctor–patient relationship, named the model of mutual participation. In contrast to previous models, this is a process that demands ‘more complex psychological and social organisation on the part of both participants’.^4^ The patient-centred approach and patient involvement are now core dimensions of what is considered high-quality health care.^5^ This corresponds to the visions of general practice in Denmark, where the patients’ needs form the pivotal point of GPs’ work.^6^

Likewise, the CCM describes the patient being ‘informed and activated’ as a prerequisite for ‘productive interactions’ with a prepared, proactive practice team to reach improved outcomes.^1^ CC consultations differ from other types of general practice consultations because they most often follow a guideline-oriented agenda with a focus on somatic and biomedical aspects of the specific conditions, however, studies have shown that patients’ agendas might be downgraded in this context.^7^ A Danish observational study showed that there is a significant risk that concerns voiced by patients were missed in CC consultations.^8^

The prevalence of patients with multimorbidity is increasing and adds challenges to CC regarding clinical guidelines, polypharmacy, and organisation of care.^9^ Moreover, GPs experience some patients as complex because of an aggregation of diagnoses, advanced age, frailty, and social, cultural, or economic factors.^10,11^ Many patients with multimorbidity are in a vulnerable position because of the burden of disease often with complex psychosocial needs^12^ including limited life opportunities, which we define as complex health conditions in the present study. A Scottish study showed that patients in deprived areas had less desire for shared decision making and perceived their GPs as less empathetic compared with patients in affluent areas.^13^ From a GP perspective, the patients’ challenges may manifest as struggles with adhering to the treatment regimen outlined in guidelines,^14^ which easily challenge the collaboration between the GP and the patient. Collaboration may refer to different types of alliances, supporting collaborative interaction, and relationships to different degrees.

To understand possible tensions in the CC partnership between GPs and patients with complex health conditions, it is important to explore how they both experience the challenges in the attempts to collaborate and maintain an alliance. Therefore, this study focuses on dyads of patients and GPs, where collaboration is troubled and the recommended treatment fails. The aim is to explore how dyads of GPs and patients, where GPs deem the patient has complex health problems and difficulties following treatment, perceive challenges in their CC partnership and how they manage these.

## Method

### Design, participants, and data generation

Twelve GPs were recruited from two rural municipalities in Denmark characterised by a high prevalence of chronic conditions and lower socioeconomic status. First, researcher MATK presented the project at three local GP meetings, which resulted in four GPs agreeing to participate. Thereafter, an additional 10 GPs were consecutively invited from among 55 GPs in the study area. To increase variation, the GPs were purposely sampled based on age, gender, practice size, and location. Of these, eight agreed to participate (Table 1) whose characteristics were similar to the rest of the GPs in the study area. Before interviews, each GP had chosen three case patients for an anonymous discussion with the following selection criteria:

- diagnosis of type 2 diabetes;
- diagnosis of ≥1 additional chronic condition;
- the GP experienced the patient having difficulty following treatment.

**Table 1.** Characteristics of the GPs in the study (*n* = 12)

Characteristic	Value
**Median age (range), years**	56 (37–69)
**Gender, male/female, *n***	6/6
**Median seniority (range), years**	16 (1–41)
**Practice size**	
1 GP	6
2–3 GPs	5
>3 GPs	1
**Practice location**
Semirural, <5000	4
Urban, >5000	8
**Distance to Hospital**
<30 minutes’ drive (range, minutes)	5 (2–27)
>30 minutes’ drive (range, minutes)	7 (35–51)

Type 2 diabetes was chosen because of its high prevalence. The case patients had prevalent health conditions often including mental disorders and addiction (Table 2).

**Table 2.** Characteristics of the 36 case patients identified by the GPs and the 12 interviewed patients

Characteristic		Case patients (*n* = 36)	Interviewed patients (*n* = 12)
**Mean age (range), years**		62.5 (37–81)	59.2 (37–72)
**Gender, male/female, *n* (%)**		21/15 (58/42)	8/4 (67/33)
**Chronic conditions, *n* (%)**			
Diabetes		36 (100)	12 (100)
Heart disease		18 (50)	6 (50)
Mental disorder^a^		16 (44)	5 (42)
Obesity		14 (39)	5 (42)
Addiction		9 (25)	5
Musculoskeletal		8 (22)	4
Respiratory disease		4 (11)	1

^a^Such as anxiety, bipolar disorder, and PTSD.

After the interview, the GPs were asked if any of the 36 patients would not be suitable for an interview, and the GPs excluded five patients due to severe somatic and psychiatric diseases, or dementia. To optimise variation in gender, age, and comorbidities, purposive sampling resulted in 17 patients being invited, of whom 14 agreed to participate. Two patients cancelled the interview because of disease exacerbation, resulting in 12 pairs of GPs and patients. We included interview participants until information power was reached.^15^

In 2015–2016, researcher and GP MATK conducted individual, semi-structured interviews with participating GPs in their clinics, and patients in their homes or at a neutral place. The interviews were audiorecorded and lasted between 50 and 75 min. A GP and a patient interview guide gave flexible frameworks for questioning and both had themes about collaboration, self-care, and chronic conditions.

### Analysis

For data analysis we used systematic text condensation, which is commonly used in Scandinavian general practice research. It is based on social constructionism that aims to derive knowledge from everyday experiences, and suited to a cross-case analysis of a phenomenon to identify new descriptions and concepts.^16^ During analysis of interviews with GPs and patients separately, we identified code groups of collaboration in both groups. Thereafter, codes of each of the GP–patient pairs were combined as cases, and finally we performed a cross-case analysis of the 12 pairs focusing on what agreement and differences between perspectives about their collaboration and relationships were of concern.

The researchers included four medical doctors, with and without GP training, and an anthropologist. The analysis was primarily performed by MATK supported by ADG and MBR Open coding by hand was used to analyse the transcripts and through comparison of these codes, the coding groups were negotiated.

## Results

In the dyads, we found that the GPs and the patients expressed specific challenges of collaboration, which was often related to the complexity of patients who were disadvantaged with a heavy disease burden, social, and addiction problems. The GPs in this study expressed the expectation that both parties would do their part to enhance the quality of care as intended in the DMPs. When the GPs experienced that patients did not contribute to the collaboration, had difficulties in following the treatment, or did not show up for regular check-ups, it gave the GP a hunch that something was wrong and that the patient might not fit into the guidelines of the DMPs. Many patients described that they valued a good relationship with the GP, where they experienced being accepted and listened to, and emphasised the mutual need for both parties to adjust and adapt to each other. The patients expected the GP to define the possible measures, but in the end, it was solely up to the patient to follow the given advice. The results section is divided into three parts: a characterisation of collaboration, identification of collaborative challenges, and how challenges were managed.

### A characterisation of collaboration: stuck or unstable

When analysing the interviews with GPs and their patients, we saw that the CC of patients with complex health conditions and difficulty in following the treatment could be characterised as either stuck or unstable.

The six stuck collaborations most often had a continued, stable contact between the GP and the patient, but with poor disease regulation. In the long run, both patients and GPs recognised considerable disease progression in terms of symptoms or complications, or in the best cases status quo, despite different attempts to improve treatment. Most consultations were about repeating lifestyle advice and addressing issues of compliance. However, lifestyle changes rarely occurred among these patients and if so, they would most often be short lasting.

Six GP–patient pairs were characterised as unstable CC primarily because of a heavy burden of somatic diseases, severe mental disorders, or misuse of alcohol or drugs. In these cases, the GPs experienced many missed appointments, failed referrals to specialised treatment, and that patients would suddenly turn up in the clinic for CC or with acute complications. If the GPs accepted the reasons for this behaviour, they would have a greater understanding of these patients compared with the patients with stable (stuck) courses. Tables 3 and 4 illustrate scenarios of stuck and unstable CC.

**Table 3.** Example of a stuck patient–GP collaboration

Thomas is a retired carpenter in his late 60s with diabetes and well-treated prostate cancer. Both his mother and sister were alcoholic and died at a younger age. He visits the GP on a regular basis and seems occupied with his health in terms of medication and monitoring blood sugar levels. However, his diabetes is badly regulated and he has significant complications in the eyes and the kidneys. The GP has tried to motivate Thomas to take better care of his diabetes in terms of eating healthier and exercising. Nonetheless, Thomas’ lifestyle is unchanged and the GP is frustrated, because the complications could have been prevented. Thomas is very happy about the collaboration with the GP. His GP feels powerless witnessing the continuing disease progression. (Dyad A)

**Table 4.** Example of an unstable patient–GP collaboration

Harry is a middle-aged business man with possible bipolar disorder and periodic alcohol overuse. He also has type 2 diabetes and chronic lower back pain. Last year, Harry and his GP had disagreements about the regulation of his diabetes. Harry thought that the GP should be able to regulate the diabetes better with new medication, but the GP felt that Harry should do more to eat healthier and lose weight. For some time, they did not talk and Harry expressed his discontent to the other staff members in the general practice, but slowly the relationship was restored and Harry decides that it would not be worth finding another GP to start it all over. However, Harry explains that he has put his diabetes completely on the shelf until the mental health stuff is in place. The GP and Harry have agreed to refer him to a psychiatrist. (Dyad I)

### Challenges in the collaboration between the patient and the GP

The GPs often experienced that their advice or treatment decisions made in stuck and unstable collaborations with the patients were forgotten or not followed. Many patients were aware of this. The GPs found that they were repeating themselves without any progression. In a stuck partnership, one experienced GP was frustrated as he believed that the serious complications could have been avoided. He explained his efforts to encourage the patient to adopt a healthier lifestyle, yet the patient perceived the disease progression as beyond his control. The patient expressed high satisfaction with the collaboration and had confidence in the GP:

*’I have cared a lot about him and tried to motivate him to change lifestyle, because he is so badly hit* [by diabetic complications] *… we have done everything possible, but he cannot be moved an inch … That’s how it is.’* (GP A)

*’I am not concerned* [about diabetic complications]*, because I have been told that it would go like this and that. Nevertheless, it is just like a path you must follow … I accept it, but I have also been informed that it could be like this.’* (Patient A)

Many GPs and patients reported that they had experienced conflicts in the consultation. In the following case, the GP made multiple attempts to encourage the patient to change his lifestyle, but the patient insisted on retaining the right to choose his own way of living:

*’He just gets so annoyed if we start talking about his diet … he is in some kind of a bubble, where he thinks that he is healthy, so he does not need to change anything.’* (GP C)

*’The thing about eating healthy, that is just not me … Moreover, I just know that I am not going to start exercising … I know what it takes and then there is nothing to do about it.’* (Patient C)

This patient expressed that he viewed the GP as a health consultant and that lifestyle was a private matter, but on the other hand, the GP saw it as a natural part of CC to keep on counselling about lifestyle.

If a conflict was unaddressed and the relationship continued, it could lead to the GP’s resignation; that is, becoming more passive and less ambitious on behalf of the patient. Consequently, the CC consultations would pointlessly replay around the same themes. In this next case, the GP reported having tried in vain and had resigned changing the patient’s lifestyle, but still intended to keep an alliance with the patient about regular consultations. The patient also lacked engagement in terms of lifestyle and experienced a lack of information:

*’Sometimes, you have to say okay, but this is your choice* [of not changing lifestyle]*. I will keep informing you and would like you to consult me. I will kindly tell you how things work, but I cannot do more – I am not a magician. I think you often hit your head against the wall, when you try your best and the patients just do something different.’* (GP J)

*’I might lose weight and I know that I could change my diet. My blood pressure would probably benefit from that, because things are somehow related. However, nobody informs me that much – they just hand out some tablets. Then I do not bother.’* (Patient J)

For the unstable courses, the collaboration was challenged if the GP did not know the reason for the shifts in collaboration. In such cases, the patients experienced a less understanding GP and could fear what would happen if the GP found out the real reason. The uncertainty made it difficult for the GP to deal with the challenges and in the worst cases, the patients avoided the GP:

*’I am frustrated that her diabetes it so poorly regulated … She often misses or comes too late for appointments. She calls us 20 times and forgets her appointments … I don’t know what is going on … ‘* (GP B)

*’I like the nurse, but I cannot stand the GP. It is because of these accusations she had about me in terms of alcohol and drugs. The GP even contacted the municipality … I was bloody furious … I don’t talk to the GP anymore. In fact, I try to avoid her.’* (Patient B)

Some patients had lost faith in treatment and believed that their health problems were unsolvable. These patients experienced being dismissed by the health system with no adequate offer of treatment or support:

*’His psychiatric disorder is probably the reason why he does not show up for the chronic care consultations … He was also referred to psychiatric treatment, but I think they gave him up, because he missed several appointments.’* (GP H)

*’What should the doctors do about my situation? They can only prescribe medicine. I have talked so much with them, and I get so tired of that.’* (Patient H)

### How challenges in stuck or unstable collaborations were managed

Conflicts were sometimes solved, which improved the relationship between the GP and the patient. Below is an example of a conflict, which was addressed and solved through shared partnership. Both parties found that it resulted in a stronger relationship:

*’He recently said it again: you are educated and you must take care of my weight loss … Then I answered: yes, and with my education, I can tell you what the wise thing is to do, but the task is yours.’* (GP I)

*’The GP and I had our fights … after all, we are a good match, because we are both tough … I was not interested in finding another GP and starting it all over … So, we had to adjust to one another.’* (Patient I)

Other conflicts were unsolved. They could remain active, or they could turn passive and unspoken. In the latter situations, it would affect the relationship negatively. Sometimes both parties were aware, but other times only one of them moved on. In this case, the GP recognised the conflict but misunderstood what it was about and thought that it was solved, because the diabetes was managed and the patient seemed happy. However, the patient avoided talking about her emotions, because the GP did not recognise the patient’s anxiety:

*’When we first met, she told me that her diabetes was all fine … then I told her that it was not regulated well, and it came as a total shock to her. She did not have a clue about that, it was so poor, and she went totally down … ‘* (GP K)

*’I tried to talk to the GP about anxiety several times … the last time, she told me that I should stop being such a pessimist … when she said that I could have hit her … Since then, I have talked to nobody about it, because I don’t want to expose myself for such a reaction again.’* (Patient K)

In some cases, the GP gave the patient some extra attention or service and thereby strengthened the relationship and trust. This GP experienced a successful change in lifestyle but found it fragile and therefore gave the patient special access. The patient recognised the exceptional partnership and appreciated that the GP understood his challenges well:

*’When I realised that alcohol was such a big problem, we talked about it and he managed to change … if he needs to talk to me, he usually gets an appointment with short notice … ‘* (GP F)

*’I have great faith in my GP, and we get along well. She always has time for me, so I really appreciate our relationship … I do not consult the other GPs, because I would rather wait for her … She knows the full picture.’* (Patient F)

Some GP–patient pairs had a shared and honest understanding of the systemic challenges in CC, and that helped either to bypass the challenges or to put up with them. Even in situations where problems were insoluble and the situation was hopeless, the support of the GP was highly valued:

*’In her case, we need other options in the health system … Due to her overweight and the wheelchair, she cannot get the right help to exercise and maybe even get back on her feet … Despite all this, she has managed to lose some weight.’* (GP G)

*’I have lost all hope in the future … I don’t mind trying to lose weight and exercise, but they gave up my training … They said that they could not help me … My GP still hopes to get me on my feet again … She supports me very much. She is always ready to listen to me.’* (Patient G)

In other cases, the GP was willing to accommodate the challenges in the relationship. Then the patients could experience a special bond with the GP – sometimes this was supported by common experiences of a difficult social situation or serious exacerbations of the chronic conditions:

*’He has his own ideas about how things should be and I think that you just have to adapt to his ideas and hope to keep him going … He has more serious problems than other patients* [alcohol and anxiety] *… His mental health is in the way of his diabetes.’* (GP D)

*’In fact, I look forward to seeing my GP … She is very thorough and so nice to me … She always has time for me, and sometimes she even laughs at me saying "oh come on" … She appreciates that I am honest with her.’* (Patient D)

Thus, the restoration of the doctor–patient alliance could happen through solving conflicts, adjusting to the patient’s needs, continued support despite unsolved problems, and containing the challenges in the relationship. These management attempts seem useful and common to both unstable and stuck relationships. If the GP sticks to a fixed biomedical focus on improving disease regulation the relationship seems not to be improved. When facing insoluble problems, the GP has an important role in maintaining the doctor–patient relationship by paying attention to the patient’s needs and as support for the patient.

## Discussion

### Summary

To our knowledge, this is the first study to explore experiences of CC through dyads of GPs and patients with complex health conditions. Overall, the patient–GP collaboration could be characterised as either stuck or unstable. In both types, the challenges were identified as pointless consultations, conflicts about lifestyle, resignation, concealment of information, and hopelessness. These challenges could be managed by solving conflicts, adjusting to the patient’s needs, accommodating the challenges in the relationship, and offering continued emotional support even for unsolved medical problems.

### Strengths and limitations

By interviewing patient–GP dyads, the perceptions and reflections of both parties can be explored in a way that would be impossible in an observational study, and this provides potential insight into the collaboration and relationship between the two partners, their understanding about each other, of diseases, priorities, ideas about care, and where the narratives clash. The high participation rates, both around 80%, allowed for successful purposive sampling that secured variation and a richness of the narrative material.

The study design depended on GPs identifying patients with complex health conditions. We believe that this contributed to the high patient participation rate. Before the GP interview, the GPs were unaware that the patient cases could be interviewed and therefore did not affect the selection of case patients before the GP interview.

To reduce the risk of conceptual blindness inherent in peer interviewing, the research team included researchers from outside general practice throughout the study.^17,18^

The study area was economically disadvantaged and rural, which might imply limited transferability to urban or more affluent settings. However, a UK study found that GPs and patients in a wide range of socioeconomic settings experienced similar challenges with multimorbidity,^19^ suggesting that the challenges experienced in this study are more likely because of dealing with complex needs, rather than social deprivation. Although we collected data in 2015–2016, we still find the results transferable, because the structure of CC and socioeconomic inequity are unchanged.

### Comparison with existing literature

This study adds to the existing knowledge that although care of patients with complex health problems might not solve the biomedical problems, the GP can make an important difference to the patient by solving conflicts, adjusting to the patient’s needs, continuing support, and by being open for exploration of emotional problems. It highlights the relational dimension, also known as the therapeutic alliance,^20^ of these patient–GP partnerships as crucial for both parties. A qualitative study of patients and their GPs about how healing relationships are developed and maintained^21^ identified three relational outcomes: trust, hope, and a sense of being known. These outcomes are highly dependent on relational continuity,^22^ but this aspect of patient care is not included in the DMPs’ emphasis on the patient–doctor collaboration. Nonetheless, prioritising the long-term relationship seems to be an important strategy for GPs when managing patients with multimorbidity for the numerous gains this can bring about such as mutual trust, deeper insight into a patient’s unique circumstances, and useable knowledge of each individual’s capacity for the tasks of illness and goals for life.^10^ A study of patients with diabetes found a positive effect on disease regulation when the patients could decide the treatment goals.^23^

As our study shows, the patient–GP partnership was often challenged by conflicts or frustrations related to being stuck or unstable. If conflicts were solved or bypassed by changing the agenda, it seemed to strengthen the relationship. This underlines the importance of addressing the patient’s agenda in CC consultations, which is fundamental for the doctor–patient relationship in general practice.^24^ Previous research showed that patients valued CC continuity even higher than clinicians^25^ and this mattered particularly for patients with complex health problems such as multimorbidity or reduced ability for self-care.^19^ Moreover, a recent Norwegian study showed that continuity in GP care reduced morbidity and mortality.^26^ CC is often shared between a GP and a nurse, but this might be inappropriate, when patients have complex health problems, because relational continuity implies familiarity and mutual confidence that can, and usually does, arise for repeated contact over time.^22^

The GPs in our study found that the DMPs did not fit the patients with complex health problems in relation to the expectation of being informed and activated,^27^ which is one of the basic assumptions of the CCM: that most patients can learn to manage their disease in terms of diet, exercise, self-measurement, and medication use. In this study, many patients struggled with substance misuse, mental, and social problems. We also found that many conflicts and frustrations in the GP–patient partnerships had to do with a lack of services for these patients. However, in its original form the CCM included a care manager for the patients with the most complex needs to serve as extra support and a link to the healthcare system. When DMPs were introduced in Denmark, this part was omitted and later evaluations concluded that there are no special services for a large group of patients with complex needs or increased vulnerability.^28^ This remains a missing link in providing services for people with CC needs.

### Implications for research and practice

This study provides insights into improving patient–GP relationships that seem stuck or unstable. We found that although GPs experience that care of patients with complex chronic conditions might not solve their biomedical problems in the short run, GPs can make an important difference to the patient by solving conflicts, adjusting to the patient’s needs, continuing support, and by being open for exploration of emotional problems. The results challenge the DMPs’ biomedical approach and reinforces the value of continuity and the personal patient–doctor relationship in general practice that in the long run might lead to significant health benefits.

We suggest that more research is needed to explore ways to strengthen the partnership between GPs and patients with complex chronic conditions. Moreover, there is a need to explore and test initiatives that provide more proactive and empowering health care for these patients, which remains an insufficiently implemented part of the CCM.

## Data

The dataset relied on in this article is available from the corresponding author on reasonable request.
